# Postoperative Hallucinations or Reality: Can Patients Differentiate Between Hallucinations and Reality?

**DOI:** 10.7759/cureus.79986

**Published:** 2025-03-03

**Authors:** Hideki Onishi, Akira Kitamura, Seika Oono, Yoshitaka Ooya, Mayumi Ishida

**Affiliations:** 1 Department of Psycho-oncology, Saitama Medical University International Medical Center, Hidaka, JPN; 2 Department of Anesthesiology, Saitama Medical University International Medical Center, Hidaka, JPN; 3 Department of Emergency and Acute Medicine, Saitama Medical University International Medical Center, Hidaka, JPN

**Keywords:** delirium, hallucinations, postoperative delirium, sexual hallucinations, visual and auditory hallucinations

## Abstract

Delirium is the most common psychiatric complication after surgery and is known to be a source of distress for patients. During the course of postoperative delirium, patients may experience hallucinations and can sometimes confuse these hallucinations with reality. We present a patient who experienced vivid hallucinations in the ICU after surgery that were indistinguishable from reality. The patient, a woman in her 20s, was admitted to the ICU after surgery and was confused because she felt that other patients had made sexual comments to her. However, the patient was able to recognize them as hallucinations because, despite her extreme myopia, she could clearly see her surroundings. A detailed examination of the medical records allowed the medical team to also determine that they were hallucinations. Healthcare professionals should be aware that patients may experience hallucinations that are indistinguishable from reality.

## Introduction

Delirium is the most common psychiatric complication after surgery, experienced by approximately 30% of patients [1], and is known to be a source of distress for both patients and their families [2]. During the course of postoperative delirium, patients may experience hallucinations, with visual hallucinations being the most common form [3], and can sometimes confuse these hallucinations with reality. In some cases, this has led to situations in which patients have alleged sexual assault by medical staff, resulting in legal action [4]. Therefore, it is essential to expand our knowledge regarding the nature of the hallucinations experienced by patients, as well as strategies for their successful management. We present a patient who experienced vivid hallucinations in the ICU after surgery that were indistinguishable from reality. However, the patient was able to recognize them as hallucinations, and a detailed examination of the medical records allowed the medical team to also determine that they were hallucinations.

## Case presentation

The patient was a female in her 20s with no history of mental illness or substance abuse who was diagnosed with maxillary sinus osteosarcoma and underwent tumor resection. General anesthesia was administered. Induction involved the administration of 200 μg of fentanyl, 130 mg of propofol, and 50 mg of rocuronium bromide, followed by tracheal intubation. During surgery, anesthesia was maintained with 4% desflurane and remifentanil at 0.1-0.2 μg/kg/min, with additional doses of fentanyl administered as needed.

During anesthesia, the patient's hemodynamics remained stable with a blood pressure of 100/60 mmHg, a heart rate of 60-70 bpm, and an SpO2 of approximately 99%. The intraoperative blood loss was 450 ml, with a blood hemoglobin level of 9-10 g/dl. The surgery lasted 18 hours and 55 minutes, and the total anesthesia time was 20 hours and 21 minutes. Postoperatively, the anesthesiologist confirmed that the patient had recovered from anesthesia, and she was admitted to the ICU. The patient (the timing of the incident after admission to the ICU is unclear) later reported that the patient in the bed two beds away on her left (approximately four meters away and oriented in the same direction) made sexual remarks, saying, "I can see your breasts."

Initially, she was confused because the events unfolding before her seemed so vivid that she thought they were real. However, she realized that she was extremely nearsighted (her visual acuity is 0.03) and would not be able to see several meters ahead without glasses. Despite this, she could see her surroundings in the ICU clearly, and this allowed her to understand that she was experiencing hallucinations. Examinations revealed no central nervous system (CNS) abnormalities, and there were no abnormalities observed on blood and biochemical tests that could induce hallucinations. Moreover, during the ICU stay, the patient was examined by the anesthesiologist and ICU doctors, but no particular abnormalities were noted. According to the patient, what she was experiencing seemed so real that she could not distinguish between the hallucinations and reality.

**Figure 1:**
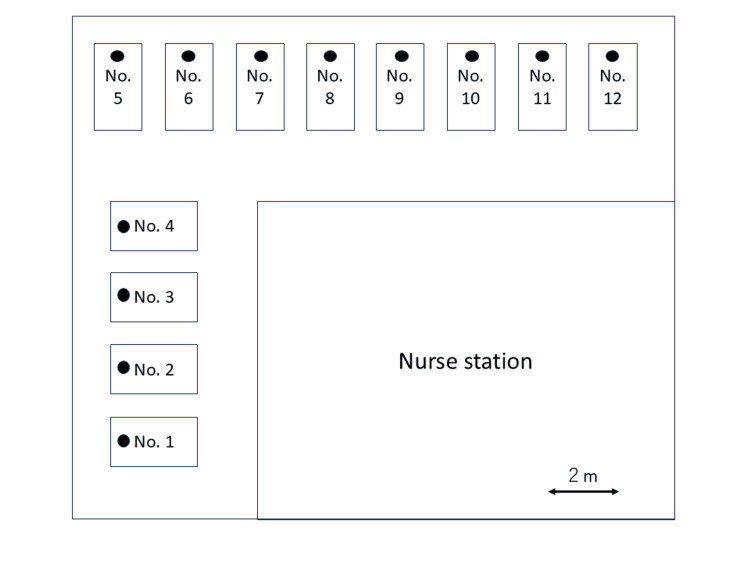
Bed layout in the ICU The position of the patient’s head is denoted by a filled circle (●). The beds were separated by a distance of 2 meters

Regarding the sexual remarks, the authenticity of which was uncertain, we checked the position of her bed and the bed two places away on her left on the electronic medical records. This confirmed that she was in bed no. 4 and that there was no bed two places away from hers on the left and oriented in the same direction (Figure 1). This further verified that what she experienced was indeed a hallucination.

The ICU staff's chart notes indicate that she had been in the ICU for 24 hours but was calm during her stay, with no recorded signs of problematic behavior, such as hallucinations or agitation. Subsequent follow-up revealed no episodes similar to those observed after ICU admission, and no CNS abnormalities were noted.

## Discussion

This case illustrates that the hallucinations associated with delirium are particularly vivid and that patients may have difficulty in distinguishing such hallucinations from reality. In the present case, the patient experienced vivid hallucinations and initially could not distinguish between reality and the hallucinations. However, she recognized that they were hallucinations when she realized that her severe nearsightedness prevented her from seeing more than a few meters without her glasses. Additionally, upon investigating the location of the patient’s bed in the electronic medical records, it was found that the bed of the man she claimed had sexually harassed her did not actually exist, allowing the staff to conclude that this too was a hallucination.

However, if the patient were not nearsighted and if a patient had actually occupied the relevant bed, there would need to be clear denial from the staff, who were present at the time, stating that such an incident did not occur. Without such, due to the vivid nature of the hallucinations, there is a risk that the patient could mistakenly believe they experienced sexual harassment from another patient.

Additionally, while the subject of the hallucinations experienced by the patient in this case was another patient, it is also possible for healthcare providers to become the subject of hallucinations. There have been instances where healthcare professionals were the subjects of sexual hallucinations, leading to legal action [4,5]. To avoid misunderstandings by patients, medical staff should remain vigilant regarding the patient's level of consciousness and hallucinations. If such conditions similar to those in the present case are observed, it may be necessary to ensure that patient care and nursing are not carried out alone whenever possible.

Although the patient experienced vivid hallucinations, there were no notes relating such experiences in the medical records. It is not uncommon for patients with hypoactive delirium to appear calm at first glance, and reports indicate that about 30% of patients with hypoactive delirium experience hallucinations [6].

## Conclusions

In conclusion, it is important for healthcare providers to be aware that patients may experience vivid hallucinations due to postoperative delirium, and these experiences may be perceived as reality. Such experiences can be distressing and, in some cases, may develop into conflicts with healthcare providers or other patients. Additionally, even if a patient appears calm, they may still be experiencing vivid hallucinations, making it essential to regularly check for delirium and hallucinations.

The patient's realization, in the present case, that what she was experiencing was a hallucination suggests that distinguishing between hallucinations and reality can be facilitated by examining the patient's vision. Additionally, investigating the patient's bed position using the electronic medical record system may also aid in differentiating between reality and hallucinations. To prevent the patient from confusing hallucinations with reality and to better protect healthcare providers, a multifaceted approach is necessary to distinguish between hallucinations and reality.
